# Virtual Nature as an Intervention for Reducing Stress and Improving Mood in People with Substance Use Disorder

**DOI:** 10.1155/2020/1892390

**Published:** 2020-05-19

**Authors:** Lori Reynolds, Oaklee Rogers, Andrew Benford, Ammie Ingwaldson, Bethany Vu, Tiffany Holstege, Korinna Alvarado

**Affiliations:** ^1^Department of Occupational Therapy, Northern Arizona University, 435 N 5th Street, Phoenix, AZ 85004, USA; ^2^Faculty Lead for the Community Health Mentor Program (CHMP), 435 N 5th Street, Phoenix, AZ 85004, USA

## Abstract

Substance use disorder (SUD) afflicts a large percentage of the United States population, with negative implications that cost more than $420 billion annually. This population often experiences negative emotions throughout the recovery process, including anxiety, depression, stress, and negative affect. Currently, evidence-based treatment strategies for SUD include cognitive behavioral therapy, motivational interviewing, 12-step programs, and mindfulness-based treatment. One intervention that has not been studied at length among individuals with SUD is use of the natural environment as treatment. Among other patient populations, nature has been shown to reduce stress and anxiety by regulating autonomic nervous system function, reducing symptoms of depression, and improving mood. The purpose of this study was to investigate whether viewing nature videos could similarly reduce stress and improve mood in individuals with SUD. A crossover design was used to compare viewing a nature scene and practicing mindfulness-based activities for women with SUD at a residential treatment facility. Over four weeks, participants engaged in the two activities for the first 10 minutes of their daily program. Immediately before and after each 10 minute session, measures were taken for heart rate, in beats per minute (BPM); affect, using the Positive and Negative Affect Scale (PANAS); and overall mood, using a 10-point rating scale from “very unpleasant” to “pleasant.” Thirty-six women completed the study. For viewing a nature scene and practicing the mindfulness-based activities, there were statistically significant reductions in mean negative affect scores (*p*=0.001) and heart rate (*p* ≤ 0.001). In addition, for participants in both conditions, overall mood improved significantly (*p*=0.030). The results from this study provide initial evidence that viewing nature has similar benefits to MBT in the treatment of stress and negative mood associated with the SUD recovery process and may be an additional, cost-effective treatment strategy for individuals with SUD.

## 1. Introduction

Substance use disorder (SUD) afflicts a large percentage of the population, with negative implications that cost the United States more than $400 billion annually [1, 2]. Individuals with SUD may seek treatment from different healthcare providers in various settings, but only 1 in 10 receive any form of specialty treatment for their disorder. Treatment for SUD often includes behavioral, social, and cognitive interventions to address psychological and emotional effects, in addition to pharmacological interventions [3]. Individuals who participate in treatment for a prolonged period, such as in a residential treatment program, are more likely to stop using substances, reduce criminal engagement, and improve their ability to function [4]. Although abstaining from using a substance and recovery are possible, the process to get there is not an easy one [5].

According to the recent Surgeon General's Report, more than 60% of those who receive treatment for addiction relapse within the first year indicate the complexities and challenges of the recovery process [6]. Throughout the recovery process, individuals with SUD often experience negative emotions, including anxiety [7–10], depression [11, 12], stress [8, 10, 12], and negative affect [9, 11, 12]. Individuals with alcoholism have increased negative affect, disappointment, and feelings of worthlessness and decreased positive affect that cause cravings to use alcohol for emotional regulation [11]. Those with SUD and a mood or anxiety disorder have a significantly increased craving intensity with an associated frequency of substance use and risk of relapse [7]. Knowing the range of emotions and physiological changes that individuals go through during their recovery process, especially when it is compounded with another mental illness, highlights the need to use evidence-based treatment methods to assist in the recovery process [7].

Currently, nonpharmacological evidence-based strategies to treat SUD include cognitive behavioral therapy (CBT) [13, 14], motivational interviewing [15, 16], 12-step programs [17, 18], and mindfulness-based treatment [19, 20]. Over the last several years, various forms of mindfulness-based therapy (MBT) have been increasingly implemented in SUD recovery treatment programs [21]. MBT strategies are associated with improved emotional regulation and mood, reduced stress, and reduced misuse of substances to a greater extent when compared with educational programs, supportive therapy, relaxation, imagery, and art therapy [12, 19, 21–24]. Hofmann and Gómez [25] found the effects of MBT for anxiety and depression to be comparable to CBT. Studies show that mindfulness-based cognitive therapy (MBCT) contributes to non-pharmacologic treatment approaches, reduces dysregulation of affect and risk of relapse of major depressive symptoms, and significantly reduces substance use [26, 27, 28]. Like other MBT approaches, mindfulness-based relapse prevention (MBRP) has similar benefits of improving emotional affect, mood and stress. In addition, compared with usual treatment, MBRP is associated with greater reduction in frequency of use, cravings, and withdrawal symptoms, and significantly lower expectancies of drug use [20, 29–32]. In one study, MBRP outperformed 12-step and psychoeducational treatment strategies in their ability to reduce substance use [33]. The research surrounding the use of MBT in treatment of SUD suggests that it is a promising non-pharmacological approach to care in a variety of ways.

Similar to the benefits of MBT, among the general population, there is a large body of research on exposure to nature as an intervention for reducing stress, anxiety, agitation, and depression, as well as for improving mood [34–37]. This research is rooted in stress reduction theory, which suggests that exposure to nature reduces stress through activation of the parasympathetic nervous system, which is facilitated by humans' innate and evolutionary preference for natural environments [38]. Improvement in mood and reduction in stress have been found with both exposure to actual nature and to images of nature (e.g., pictures, slideshows, videos, and virtual reality). In some studies, individuals are exposed to a cognitive/emotional stressor, and after exposure to actual or images of nature, stress is reduced as indicated by reductions in heart rate and blood pressure and by parasympathetic response, which is measured by heart rate variability [4, 34, 39–41]. Changes in mood are measured with various standardized scales and inventories such as the Cohen-Mansfield Agitation Inventory, the State-Trait Anxiety Inventory, and the Positive and Negative Affect Scale (PANAS). The improvement in mood among the general population is also seen among individuals with dementia. The majority of people with dementia experience negative emotions/affect such as agitation and/or anxiety that are accompanied by behaviors difficult for care staff to manage and that create stress for the individuals experiencing the emotions [42, 44]. As with the general population, direct exposure to nature has been shown to reduce the negative emotions/affect associated with dementia [34, 39]. For example, studies have demonstrated that agitation is significantly reduced relative to time spent in outdoor gardens [44, 45]. In several studies, reduced stress and improved mood were achieved in as little as 10 minutes of viewing nature [35, 39, 40].

To date, research on the mental health benefits of nature has been conducted among the general population and individuals with dementia. Individuals with SUD often experience stress and negative emotions as part of the recovery process; however, research on the use of nature as a treatment with this population appears to be limited to wilderness-based SUD programs [46–49]. In one qualitative study conducted in a natural environment, individuals 20–50 years old reported enjoying the peace, quiet, and fresh air of being in nature, as opposed to the typical treatment environment which was perceived as chaotic and intense. In another study, participants reported enjoying interacting with wildlife [50]. In these studies, treatment has focused on activities completed in nature rather than on the direct benefits of nature itself.

Given the stress and negative emotions experienced by individuals recovering from SUD and the large body of research supporting the mental health benefits of nature, it is important to examine whether these benefits could also be experienced among this population. Therefore, the purpose of this study was to investigate whether viewing a nature video could reduce stress and negative emotions among women in a residential SUD treatment program, after 10 minutes of exposure, as has been demonstrated with other populations.

## 2. Methods

A crossover design was used to compare a virtual nature condition with typical programming using MBT strategies. A control condition could not be used because services could not be withheld from individuals in the residential treatment program. Approval was received from Northern Arizona University's institutional review board office to conduct this research study.

### 2.1. Setting

The study was conducted in a residential SUD treatment facility, located in the Phoenix Valley, that serves only women aged 18 years and older (60% being 18 to 35 years of age) and offers three phases of treatment. The first phase is a highly structured and intensive program that includes peer mentoring, educational programming, 12-step meetings, SMART recovery groups, and counseling. The second phase includes similar programming as the first phase but focuses more on community reintegration, employment services, and higher-level life skills. The third phase is an outpatient program that further develops the previous learned skills to continue to work towards recovery and wellness. All participants in the first phase of treatment were invited to participate in the study due to the average length of time at the facility, likelihood of relapse to occur in the first phase, and structured nature of the phase.

### 2.2. Population

The study included women who were 18 years and older and enrolled in the first phase of the SUD treatment program. Exclusion criteria were women under the age of 18. All interested residents who met these inclusion criteria were provided with a consent form that described the purpose of the study. Based on a similar study by [51], using a power of 0.90, a sample size was determined to be 40 participants, each with 16 data collection times in a two-treatment crossover design. Forty-five women provided consent, and five withdrew from the recovery program before the study began. Among the 40 women who provided consent, 36 participated throughout the study period. The reasons for attrition included leaving the residential treatment facility, not being at the facility during the entire length of the study or transitioning to the second treatment phase.

### 2.3. Procedures

Women in the recovery program started each day in a preassigned group in one of three large rooms with their peers and at least one staff member. Each morning, at the start of programming, participants either viewed a nature video or engaged in an MBT activity for the first 10 minutes of their daily programming. MBT activities are typical programming for the recovery program. Over the four-week study, participants were exposed 2 days a week to a nature video with natural sounds and 2 days a week to MBT. The order of these exposures varied each week for each of the 3 groups of participants (Table 1).

**Figure 1:**
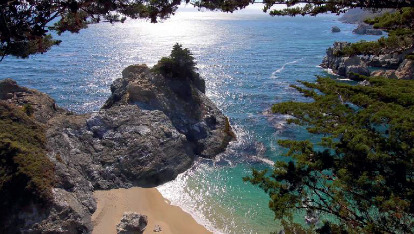
Video image.

The MBT condition consisted of 10 minutes of activities, such as sitting quietly to meditate, or listening to a guided meditation, or a reading from the staff. These activities varied based on the staff member's plan; however, each session was consistent with standard MBT techniques. In the nature video condition, participants viewed a high-definition, fixed-angle nature video with naturally occurring sounds. The same video was shown throughout the study. The video displayed a cliff-top view overlooking the Pacific Ocean with trees in the foreground, a waterfall coming off a cliff, seabirds emerging in and out of view, and naturally occurring sounds from the scene (Figure 1). Participants were instructed simply to remain quiet throughout the 10 minutes and to sit within direct view of the video.

Prior to and immediately after 10 minutes of viewing the nature video and participating in the MBT activities, the participants' heart rate was taken using a pulse-oximeter and mood was assessed using the 10-item self-report PANAS [52] and a self-report rating of overall mood on a 10-point scale from very unpleasant (1) to very pleasant (10). The 10-item PANAS measures positive affect for interested, excited, strong, enthusiastic, proud, alert, inspired, determined, attentive, and active. Negative affect is measured for distressed, upset, guilty, scared, hostile, irritable, ashamed, nervous, jittery, and afraid.

Researchers trained the staff to monitor participants' effectiveness in taking and logging heart rate and assessing and rating overall mood, before and after each condition. At the start of the study, the researchers and research assistants were present at the beginning and end of the 10-minute period to support staff and help ensure completeness of data collection. The supervision by researchers and research assistants was faded over time as staff demonstrated competence in assisting participants in completing the research protocol.

### 2.4. Data Analysis

Summary descriptive statistics were calculated as means (standard deviation) and counts (percentages). Model-based statistics were calculated as means (standard error of the mean). A generalized estimating equation approach was used to evaluate differences in heart rate, negative and positive affect, and overall mood assessed before and after each condition as well as between conditions. The interaction between pre- vs postcondition measurements and condition type (nature vs MBT) was of primary interest; however, main effects were also of interest. An alpha of 0.05 (two-tailed) was used as the criterion for statistical significance. SPSS ver. 25 was used for the analyses.

## 3. Results

Thirty-six participants completed the study, with 30 reporting their age, which ranged from 18–61 years (36.2 ± 12.7). All participants were in the first phase of treatment and had a diagnosis of SUD. As requested by the recovery program, additional sociodemographic data were not collected to respect the women's anonymity. Due to inconsistency in attendance by the participants in the research study, complete data could only be obtained for 9 conditions out of the 16 projected at the beginning of the study. Participants missed days when data were collected due to not entering the treatment facility until after the study began, attending an appointment that required them to miss the first part the group and/or leaving the treatment facility prior to the conclusion of the study. There was not a single participant who participated in all 16 days of the research study. The days missed ranged from two to nine (mode = 4 days missed, mean = 5.89 days missed).

As a result, analysis was performed using data from 9 conditions for the 36 participants. Considering the truncated number of conditions that could be evaluated, the adequacy of our sample size was reconsidered. Given the crossover design and nine repeated measurements available, 36 participants would yield .90 power to detect an effect size (f) of .16, alpha = 0.05, assuming an average correlation of *r* = 0.60 across contiguous measurements. This effect size is analogous to Cohen's *d* = 0.32, classified as “medium” [53].

**Figure 2:**
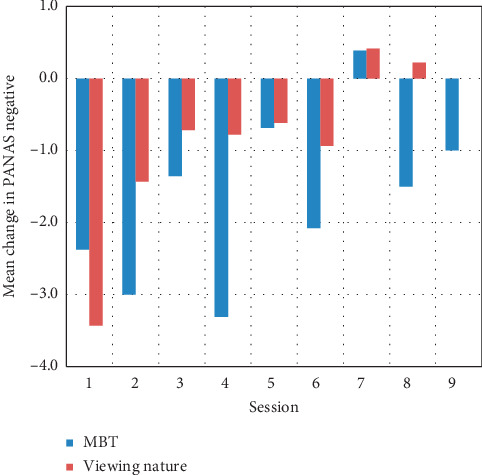
Mean change in heart rate for all participants across 9 sessions (MBT and viewing nature).

**Figure 3:**
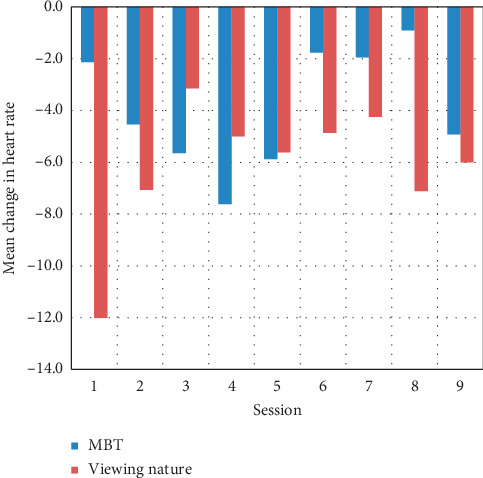
Mean change in negative affect for all participants.

**Figure 4:**
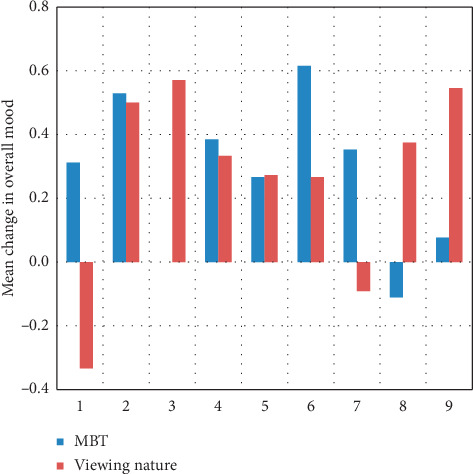
Mean change in overall mood for all participants across 9 sessions (MBT and viewing nature).

Summary descriptive statistics were calculated as means (standard deviation) and counts (percentages). Model-based statistics were calculated as means (standard error of the mean). A generalized estimating equations approach was used to evaluate differences in heart rate, negative and positive affect, and overall mood assessed before and after each condition as well as between conditions. Specifically, the change scores from pre- to postsession were calculated for each participant, and then these values were modeled by group over time. The average change scores for each session, by group, are provided (Figures 2–4). The interaction between pre- vs postcondition measurements and condition type (nature vs MBT) was of primary interest; however, main effects were also of interest. An alpha of 0.05 (two-tailed) was used as the criterion for statistical significance. SPSS ver. 25 was used for the analyses.

After 10 minutes of participation, mean heart rate decreased 3.76 BPM in the MBT condition and 5.61 BPM in the nature video condition from pre- to postmeasurement (*p* ≤ 0.001). This change did not differ across conditions (Figure 2). Analysis of mood revealed a statistically significant reduction in mean PANAS negative affect scores (*p*=0.001) in both the nature video (−0.9074) and MBT (−1.7394) conditions for pre-to postmeasurement, although no difference in change was noted across conditions (*p*=0.145; Figure 3). PANAS positive affect scores did not change significantly for viewing the nature video (mean change −0.4155; *p*=0.500) or participating in the MBT activities (mean change −0.0049; *p*=0.156); no significant difference was observed across conditions (*p*=0.229). Overall mood (pleasant feelings) improved significantly (*p*=0.030) for both conditions, with a mean change score of 0.2369 for viewing nature and 0.2938 for MBT (Figure 4).

## 4. Discussion

To our knowledge, this is the first study to be reported on the effects of virtual nature on measures of stress and mood. The results indicate potential that viewing and listening to nature videos (virtual nature) could be beneficial as an adjunctive therapy in SUD treatment programs. Key findings are statistically significant decreases in heart rate (a measure of stress reduction) and negative affect and improvement in overall mood among participants in both conditions in only a 10 minute timeframe. These results are consistent with prior research on the benefits of viewing nature for reducing stress and improving mood [34, 39, 54, 55], within a 10-minute timeframe [35, 56, 57]. The results of this study also show that viewing nature appears to be similarly beneficial to mindfulness-based strategies. While SUD recovery programs are increasingly using MBT strategies [12, 20, 21, 24], the results from this study provide initial evidence that viewing nature could be used as an additional programming strategy, with similar benefits to mindfulness-based strategies.

While the results of this study show a reduction in stress, as measured by heart rate, and negative affect, and an improvement in overall mood, interestingly positive affect did not improve in either condition. Perhaps the lack of improvement in positive mood may be explained by data collection occurring in the morning at the start programming when participants may have a higher level of positive mood and therefore less change is available to be measured. In future studies, it may be beneficial to examine the effect of conditions offered at various times of the day, to assess if a similarity or difference among conditions exists. Given the constraints of conducting the study within an existing program, measurement of mood took place in a group environment with as many as 18 other participants, with people coming into the group at varying times during pretesting and with talking among participants. This context may have influenced the mood and possibly self-reporting of mood. While previous studies have used heart rate as a measure of stress reduction [37, 38, 39, 41], a limitation of this study is that it was the only physiologic measure used. In future studies, additional measures such as blood pressure and or heart rate variability could strengthen the outcome measures. A key limitation of this study is that a control condition could not be used, as treatment could not be withheld from participants. The lack of a control condition could explain the similar outcomes for mood and heart rate among the nature video and MBT conditions and reflect similarly relaxing conditions. While a power analysis determined that 16 exposures were needed, only 9 exposures were obtained because participants missed study days for offsite appointments, entering the recovery program after the study began, or discontinuing participation in the treatment program before the study ended.

## 5. Conclusions

Despite the limitations of this study, the results with only 9 conditions demonstrate great potential for virtual nature being used as an additional nonpharmacologic treatment strategy in in SUD programs, especially those in urban settings. Given that only 1 in 10 individuals receive specialty treatment for SUD [3], use of virtual nature has a practical application as a cost-effective treatment strategy that can be readily implemented to provide an acute effect for stress reduction and improvement in mood. This treatment approach is cost-effective because it requires only a large screen high-definition television and nature videos of landscape images that have elements shown to be therapeutic. In addition, very little staff training is needed to implement virtual nature treatment, thereby maintaining its low cost. While virtual nature could be used as a treatment strategy during organized programming, it has the added benefit of being used by members of SUD programs on an as-needed basis, when struggling with some of the negative emotions that occur with the recovery process. Having a room or area set up as a “virtual nature space” would allow members of SUD programs to self-manage emotions by utilizing the space on an as-needed basis. Virtual nature used in this way could therefore become part of a host of strategies that are routinized for self-management of the recovery process. While actual nature would be preferential, virtual nature is a cost-effective option for treatment facilities, who would not be able to afford the higher costs of creating an outdoor therapeutic garden. In addition, virtual nature may benefit treatment facilities located in urban areas, where land is at premium, and access to an outdoor space—for example, to create a therapeutic garden—is not available.

Creating a virtual nature space, utilized as a stand-alone, self-selected treatment strategy or integrated into programming, provides SUD recovery program participants with a routinized treatment tool that can be used to sustain stress reduction and improve mood after discharge and throughout their recovery. The prevalence of nature videos available through various online media sources make this a viable option. With knowledge of the benefits of nature, individuals in recovery can combine viewing virtual nature with direct contact with nature through visits to area parks to help sustain recovery; both experiences have evidence to support their mental health benefits.

## Figures and Tables

**Table 1:** Crossover design for one week.

*Room 1*					
Monday	Tuesday	Wednesday	Thursday	Friday	Saturday
Nature viewing	MBT	Nature viewing	MBT		

*Room 2*					
Monday	Tuesday	Wednesday	Thursday	Friday	Saturday
	MBT	Nature viewing	MBT	Nature viewing	

*Room 3*					
Monday	Tuesday	Wednesday	Thursday	Friday	Saturday
	Nature viewing		MBT	Nature viewing	MBT

Note: each week, for the 4 weeks, the order of exposure and days of week in which they occurred varied.

## Data Availability

The data used to support this study can be made available from the corresponding author upon request.
